# NLRP3 inflammasome-mediated disruption of mitochondrial homeostasis in alveolar macrophages contributes to ozone-induced acute lung inflammatory injury: NLRP3 inflammasome in AMs is involved in O _3_-induced lung injury

**DOI:** 10.3724/abbs.2024171

**Published:** 2024-10-15

**Authors:** Xinyi Miao, Xinling Li, Pengwei Ma, Mengyuan Li, Yuting Jiang, Pengpeng Wang, Xiaolei Zhou, Ling Wang, Pingping Shang, Qiao Zhang, Feifei Feng

**Affiliations:** 1 Department of Toxicology College of Public Health Zhengzhou University Zhengzhou 450001 China; 2 Department of Occupational and Environmental Health College of Public Health Zhengzhou University Zhengzhou 450001 China; 3 Department of Pulmonary Medicine Chest Hospital of Zhengzhou University Zhengzhou University Zhengzhou 450000 China; 4 Faculty of Medicine Macau University of Science and Technology Macau 999078 China; 5 Key Laboratory of Tobacco Chemistry Zhengzhou Tobacco Research Institute CNTC Zhengzhou 450001 China

**Keywords:** ozone, acute lung inflammatory injury, alveolar macrophages, NLRP3 inflammasome, mitochondrial homeostasis.

## Abstract

Ozone (O _3_), a prevalent atmospheric pollutant, can induce lung injury. However, the molecular mechanisms of O _3_-induced acute lung inflammatory injury remain unclear. In this study, we investigate the abnormal changes in and molecular mechanism of mitochondrial homeostasis in alveolar macrophages (AMs) in O _3_-induced acute lung inflammatory injury mice. Mitochondria and mitochondrial reactive oxygen species (mtROS) are labeled with Mito-Tracker® Deep Red and MitoSOX Red, respectively. Mitochondrial DNA (mtDNA) in AMs from the bronchoalveolar lavage fluid (BALF) is detected via real-time PCR, and the expressions of mitochondrial fusion/fission-related and biogenesis-related proteins in AMs are determined via immunofluorescence staining. Our data show that in O _3_-induced acute lung inflammatory injury mice, the number of AMs and the protein expression of the NLRP3 inflammasome complex in the lung tissue are increased. In AMs from O _3_-exposed mice, the number of mitochondria, mtROS, and fission-related protein DRP1 are increased, but the levels of Na ^+^‐K ^+^‐ATPase, fusion-related protein OPA1, biogenesis-related protein NRF1 and mtDNA are significantly decreased. Compared with that in O _3_-exposed WT mice, lung inflammation is attenuated, especially the indicators of mitochondrial homeostatic imbalance in AMs, which are alleviated in NLRP3 ^‒/‒^ and Caspase-1 ^‒/‒^ mice after O _3_ exposure. These findings indicate that the NLRP3 inflammasome-mediated imbalance in mitochondrial homeostasis in AMs contributes to O _3_-induced acute lung inflammatory injury. This study may provide a new target for the prevention of lung inflammation induced by O _3_.

## Introduction

In recent years, ozone (O _3_) pollution has become increasingly serious, and the rapid expansion of the pollution season has led to severe pollution even in winter and spring [1]. The number of people exposed to O _3_ will increase significantly in the future under the influence of climate change. As an important air pollutant, O _3_ has been recognized as an urgent environmental issue, primarily because of its adverse effects on human health [2]. Extensive epidemiological studies have revealed respiratory system damage caused by O _3_ exposure [ 3 , 4]. Short-term inhalation of O _3_ is associated with an increased risk of hospitalization due to respiratory diseases [5]. The impact of O _3_ exposure is evident in the induction of airway hyperresponsiveness (AHR) and alveolar degradation, which can occur even within a short period of time (3 to 6 h) at an O _3_ concentration of 4.28 mg/m ^3^. Additionally, toxicological studies have shown that even low-dose O _3_ exposure can induce acute lung injury (ALI) in mice [6]. However, the molecular mechanisms of O _3_-induced acute lung inflammatory injury remain unclear.

Alveolar macrophages (AMs) constitute the primary macrophage population within the lungs, accounting for more than 90% of lung macrophages in a healthy state [7]. These cells serve as the first line of lung defense [8] and are among the few cell types that directly interact with inhaled O _3_ [9], contributing to the initiation of acute inflammatory responses. Alveolar macrophages are divided into proinflammatory (M1) and anti-inflammatory (M2) phenotypes, and their imbalance is often associated with disease [10]. M1 macrophages may participate in the pathogenesis of asthma by releasing inflammatory factors, thus aggravating lung injury and airway remodeling, while promoting the polarization of M2 macrophages may ameliorate lung injury [11]. Dysregulated activation and cell death of AMs are thought to be central to the progression of lung inflammation [12]. These processes significantly impact the development of ALI by causing the release of diverse inflammatory mediators from AMs in response to both infectious and noninfectious stimuli [13]. NLR family pyrin domain containing 3 (NLRP3), an intracellular innate immune receptor, can bind to apoptosis-associated speck-like protein containing a CARD (ASC) and Caspase1 to form the NLRP3 inflammasome [14], promoting the activation and release of the proinflammatory cytokines IL-1β and IL-18 [15]. NLRP3 and Caspase1, as the initiating and effector proteins of the NLRP3 inflammasome, may be more valuable to study. Increasing evidences indicated that NLRP3 can alleviate ALI by regulating the AMs pyroptosis [ 16, 17]. A previous report demonstrated that the NLRP3 inflammasome is predominantly localized in AMs, which is essential for the inflammatory response and plays a critical role in the development of ALI [18]. The NLRP3 inflammasome is activated by ROS, and adenosine-5′-triphosphate (ATP) is usually produced from mitochondria [19].

Mitochondria are important organelles of cells and constitute the core of immunity, and mitochondrial homeostasis is critical to the function of cells, including AMs. Mitochondrial homeostatic mechanisms include mitochondrial dynamics, metabolism and biogenesis [20]. First, mitochondrial dynamics mainly encompass fusion and fission, which are regulated by fusion-related proteins, such as optic atrophy protein 1 (OPA1), fission-related proteins, and dynamin-related protein 1 (DRP1). Notably, the imbalance between fusion and fission defines abnormal mitochondrial architecture and function. Second, mitochondria are the powerhouses of cells and supply ATP to maintain cell duplication and activities. Na ^+^-K ^+^-ATPase, an ATP-powered ion transporter on the cell membrane, is closely related to cellular excitability [21]. In addition, the third aspect of mitochondrial homeostasis is mitochondrial biogenesis, which is the process of generating new mitochondria in cells. This complex process is dependent on the peroxisome-proliferator-activated γ coactivator-1α (PGC-1α)-nuclear respiration factor 1 and 2 (NRF-1/2)-mitochondrial transcription factor A (TFAM) pathway [22] and then drives mitochondrial DNA (mtDNA) replication and integrity [20]. The three aspects of mitochondrial homeostasis are independent of each other and regulate the function and destiny of cells. Research has shown that mitochondrial homeostasis imbalance is associated with cellular senescence [20] and neurodegenerative diseases [22] and that regulating mitochondrial homeostasis may be a potential therapeutic target in myeloid leukemia [23]. However, the molecular mechanism regulating mitochondrial homeostasis is still unclear.

In this study, we aimed to investigate the changes in and mechanism of mitochondrial homeostasis in AMs in O _3_-induced acute lung inflammatory injury to provide novel insights into preventing O _3_-induced lung diseases.

## Materials and Methods

### Mice

C57BL/6J wild-type (WT) mice (SPF, 6–8 weeks old, weighing 20–25 g) were provided by the Medical Animal Center of Zhengzhou University (Zhengzhou, China), and NLRP3 ^‒/‒^ mice (SPF, 6‒8 weeks old, weighing 20–25 g) were obtained as generous gifts from Professor Aihua Zhang in Nanjing Children’s Hospital (Nanjing, China). Caspase-1 ^‒/‒^ mice (SPF, 6–8 weeks old, weighing 20–25 g) were generously donated by Professor Guangcai Duan of Zhengzhou University. They were housed at the Animal Research Center, School of Public Health, Zhengzhou University, at a temperature of 20°C–25°C. All experimental protocols were approved by the Life Sciences Approval Ethical Review Committee in accordance with the animal guidelines established by the Zhengzhou University Experimental Program (zzurb2021-06).

### O _3_ exposure

The mice were randomly divided into two groups: the filtered air (FA) group and the 1.00 ppm O _3_ exposure group (3 h per day for 1 day), with six mice per group [24]. The chosen O _3_ concentration was informed by environmental considerations. In areas with high pollution, atmospheric O _3_ concentrations can reach levels as high as 0.20 to 0.30 ppm. Compared with primates, rodents display lower sensitivity to the toxic effects of O _3_, and a rodent’s exposure to 1.00 ppm O _3_ corresponds to approximately 0.20 ppm in humans [ 25, 26]. Therefore, in our study, the O _3_ exposure concentration was set at 1.00 ppm for the mice, simulating a human’s acute exposure during peak atmospheric O _3_ pollution. Phenotyping of the mice was performed 24 h after the final O _3_ exposure.

### Physiology of the airway and lung

A small animal ventilator (FlexiVent; SCIREQ, Montreal, Canada) was used for this purpose. The forced oscillation method was utilized to assess respiratory mechanics. The mice were anesthetized via intraperitoneal injection of 1% sodium pentobarbital. Following anesthesia, the trachea was exposed, and the mice were intubated via a small animal spirometer. Aerosized acetylcholine (Mch) at concentrations of 0, 10, 25, and 100 mg/mL was subsequently administered to stimulate the mice, and the corresponding indices were measured to assess the airway response.

### Histological staining

The lungs were first fixed in 4% paraformaldehyde and cut into 5-μm slices for hematoxylin and eosin (HE) and Masson’s trichrome (MT) staining to observe inflammatory pathological changes and collagen deposition, respectively.

### ELISA assay

The collection of BALF was carried out in accordance with our previous study [24]. Total cells present in the BALF were counted under a light microscope. Recentrifugation was performed (69 *g*, 4°C, 10 min), the supernatant was discarded, and the cell pellet was coated with Rachel Giemsa stain before sorting and counting under a microscope. The percentage of macrophages was obtained by randomly counting 200 cells and then multiplying by the total number of leukocytes to obtain the number of macrophages. The quantification of total protein was conducted using a BCA kit (Bost Biotech, Wuhan, China). The levels of tumor necrosis factor-α (TNF-α), chemokine CXCL1, and the macrophage chemotactic protein MCP1 were measured using the corresponding ELISA kits (Cusabio Biotech, Wuhan, China).

### Primary AMs isolation and purification

A total of 10 mL of BALF was collected and subjected to centrifugation (156 *g*, 4°C, 10 min). The collected cells were suspended and introduced into the corresponding Petri dish, followed by replenishment with culture medium. These Petri dishes were then placed in a temperature-controlled cell culture incubator set at 37°C with saturated humidity and 5% CO _2_. After 2 h, the original medium was discarded, and the cells were gently rinsed three times with prewarmed PBS at 37°C to eliminate residual medium. This process was repeated after 2 h, and any remaining impurities were removed. The cells that adhered were identified as primary AMs.

### Mitochondrial homeostasis analysis in lung tissue and in AMs

In lung tissue, the expressions of superoxide dismutase (SOD), malondialdehyde (MDA) and Na ^+^-K ^+^-ATPase were determined via appropriate kits. Immunohistochemical staining was employed to determine variations in the expression levels of OPA1, DRP1, and NRF1. Following deparaffinization, antigen recovery, and quenching, lung sections embedded in paraffin were sealed and subjected to an overnight incubation at 4°C with rabbit anti-OPA1, anti-DRP1, and anti-NRF1 antibodies (diluted 1:100; Bioss Biotech, Wuhan, China). The sections were subsequently treated with goat anti-rabbit secondary antibodies (1:100) for 60 min at 37°C and then washed three times with PBS. Positive staining for OPA1, DRP1, and NRF1 proteins was visualized as brown coloration. Protein expression levels were measured at 200 high vision field using average optical density (AOD) with Image J software under a microscope (Olympus, Japan).

In AMs, the morphology of the mitochondria was visualized via transmission electron microscopy. MitoSOX Red staining was used to detect mitochondrial reactive oxygen species (mtROS), and MitoTracker ^®^ Deep Red FM was used to measure the number of mitochondria. Immunofluorescence staining was used to determine changes in relevant indicators in AMs. The lung slices were blocked with goat serum for 30 min first, followed by overnight incubation with rabbit anti-OPA1, anti-DRP1, anti-NRF1, anti-Caspase1 (1:100) and rabbit anti-NLRP3 (1:250) antibodies at 4°C. The next day, the slices were incubated with 1:300-FITC- and Cy3-labeled goat anti-rabbit antibodies for 30‒60 min in the dark, and the nuclei were stained with DAPI. Finally, a fluorescence microscope (Nikon, Japan) was used to observe and capture the images, and ImageJ software was used to calculate the relative expression.

### Real-time PCR

RNA extraction was performed via TRIzol reagent (Servicebio Biotech, Wuhan, China), followed by reverse transcription into cDNA via a reverse transcription kit (Vazyme Biotech, Nanjing, China). Subsequent detection was carried out via the 7500 Fast Real-time PCR System (Applied Biosystems, Foster City, USA). The primer sequences are detailed in Supplementary Table S1. The relative mRNA expression was calculated via the 2 ^–ΔΔCt^ method.

### Statistical analysis

Data are presented as the mean±SD from three independent experiments. Statistical analyses included *t* test for two-group comparisons, one-way ANOVA for multiple-group comparisons, and the LSD test for pairwise comparisons between groups. Bonferroni correction was used to correct the *P* value. Graphs and statistical calculations were performed via GraphPad Prism 8 (GraphPad Software, La Jolla, USA). *P*< 0.05 was considered to indicate statistically significant difference.

## Results

### O _3_ exposure increases acute lung inflammatory injury by increasing the number of alveolar macrophages and AM-related chemokines

**Figure 1:**
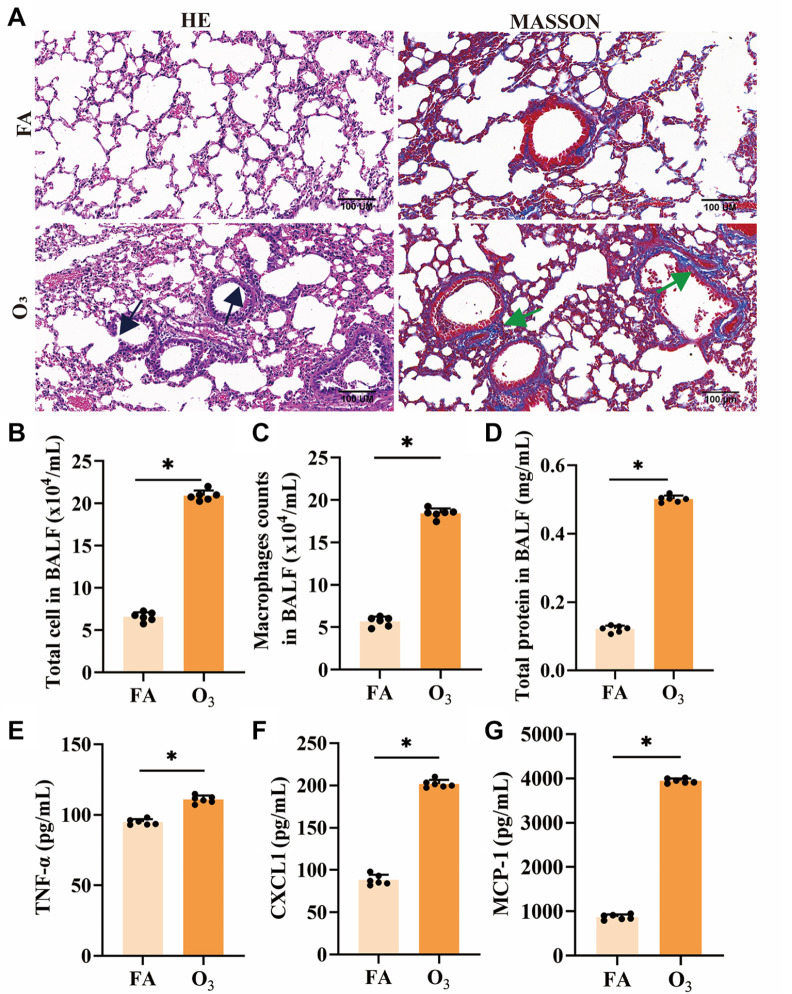
Figure 1 O _3_ exposure increases lung inflammatory injury by increasing the number of alveolar macrophages and AM-related chemokines (A) HE and MT staining of mouse lung tissue. Bronchial epithelial inflammatory cell infiltration and thickening are indicated by black arrows, and collagen deposition is indicated by green arrows. Scale bar: 100 μm. (B) Total leukocyte count in the BALF. (C) Total alveolar macrophage count in the BALF. (D) Total protein level in the BALF. (E‒G) Protein levels of TNF-α, CXCL1, and MCP-1 in the BALF of the mice. *P < 0.05.

After O _3_ exposure, inflammatory cell infiltration, thickening of the bronchial walls, and more collagen deposits in the lung tissue were observed ( Figure 1A). Furthermore, the numbers of total cells, including AMs, the levels of total protein ( Figure 1B‒D), and the levels of TNF-α and AM-related chemokines, such as CXCL1 and MCP-1 ( Figure 1E‒G), were significantly greater in the BLAFs of O _3_-exposed mice than in those of FA-treated mice. These changes are indicative of acute inflammatory injury in the lungs of O _3_-exposed mice.

### O _3_ Exposure increases NLRP3 and Caspase-1 levels in AMs

**Figure 2:**
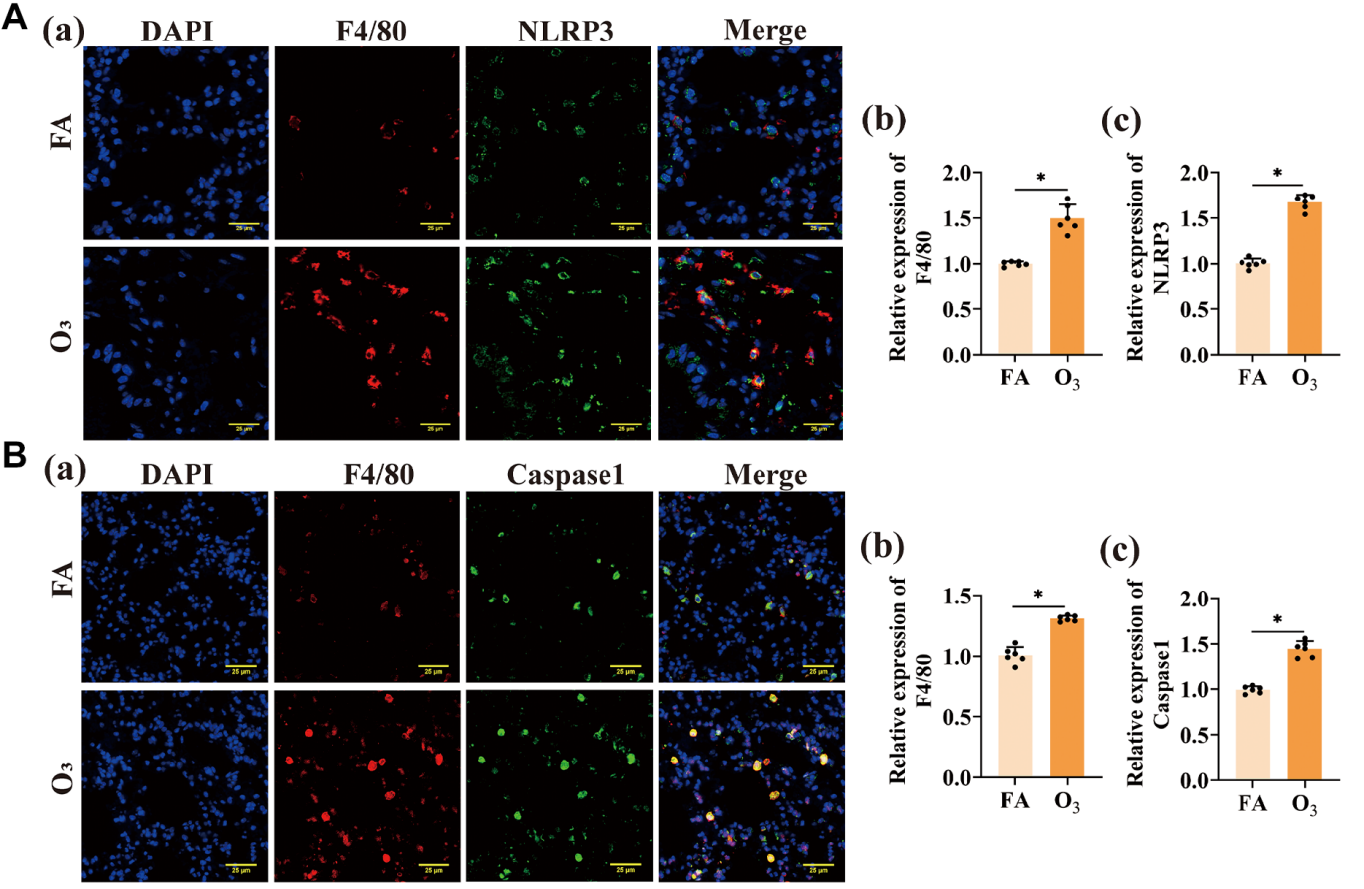
Figure 2 O _3_ Exposure increases NLRP3 and Caspase-1 levels in AMs (A) Immunofluorescence staining was used to determine the expression levels of the macrophage markers F4/80 and NLRP3 (a). Scale bar: 25 μm. ImageJ software was used to quantify F4/80 (b) and NLRP3 (c) expression levels. (B) Immunofluorescence staining was used to detect F4/80 and Caspase-1 expressions (a). Scale bar: 25 μm. ImageJ software was used for the quantification of F4/80 (b) and Caspase1 (c) expression levels. *P < 0.05.

After O _3_ exposure, immunofluorescence results showed increased expression of the marker protein F4/80 in alveolar macrophages compared to the control, indicating a discernible increase in alveolar macrophage number. Moreover, the protein expressions of NLRP3 ( Figure 2A) and Caspase1 ( Figure 2B) in AMs were elevated compared with those in the control. These findings suggest that O _3_ exposure induces an immune response characterized by alveolar macrophage activation and the activation of the NLRP3 inflammasome, potentially contributing to inflammation and lung tissue damage.

### Oxidative stress enzymes and mitochondrial fission/fusion-related proteins are altered after O _3_ exposure

**Figure 3:**
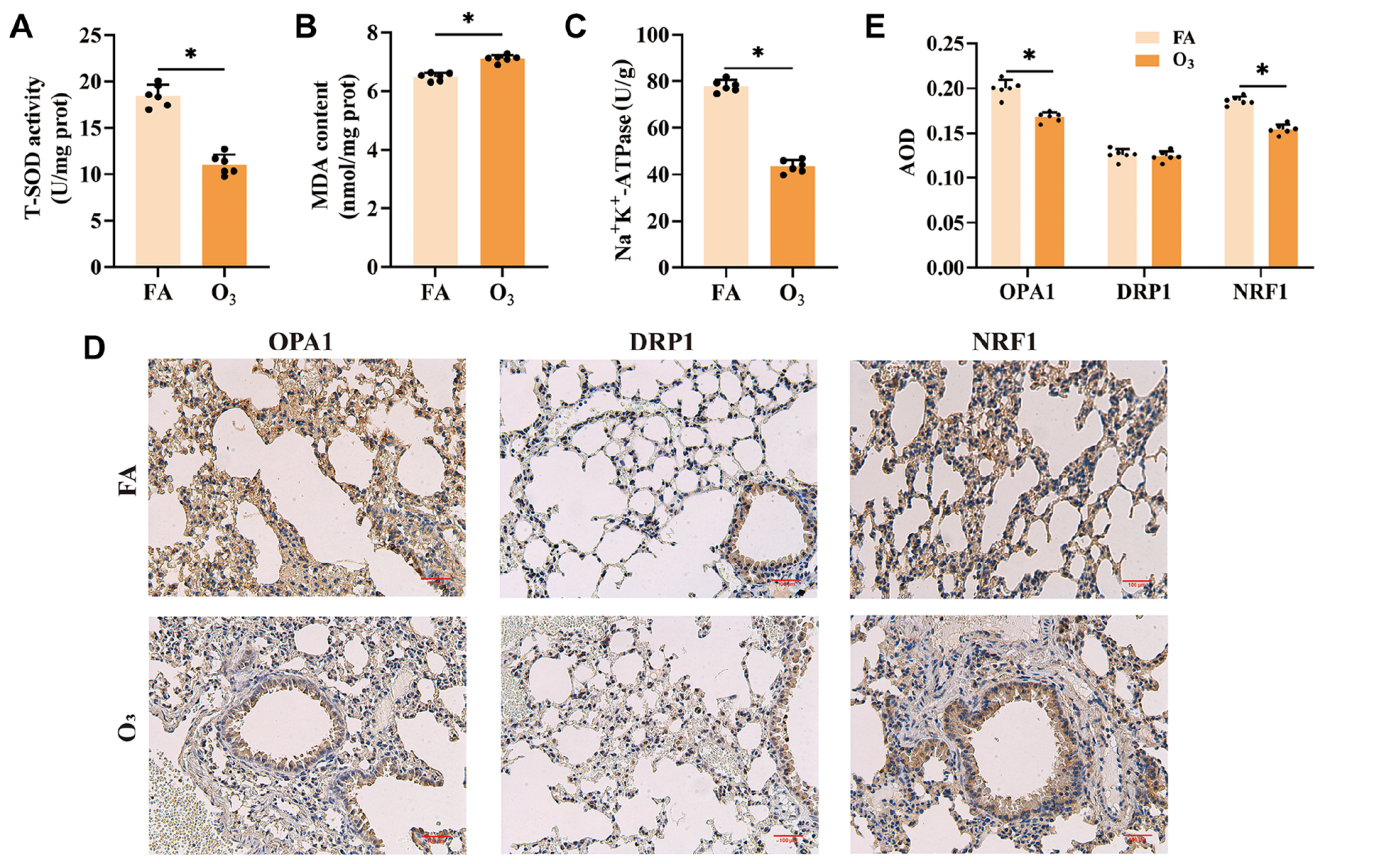
Figure 3 Activation of oxidative stress enzymes and mitochondrial fission/fusion-related proteins in lung tissue by O _3_ exposure (A-C) Measurement of SOD (A), MDA (B) and Na+-K+-ATPase (C) levels in mouse lung tissue. (D) Expressions of mitochondrial fission/fusion-related proteins were determined via immunohistochemistry. Scale bar: 100 μm. (E) Intensity measurement of mitochondrial fission/fusion-related proteins via Image-Pro Plus software. *P < 0.05.

O _3_ exposure induced changes in oxidative enzymes, as evidenced by a significant decrease in total superoxide dismutase (SOD) activity in the lung tissues of the O _3_-exposed group compared with the control group ( Figure 3A). This alteration was further confirmed by examining malondialdehyde (MDA) expression level, as depicted in Figure 3B. Notably, the activity of Na ^+^-K ^+^-ATPase, a key enzyme involved in energy conversion and mitochondrial function, was substantially reduced after O _3_ exposure ( Figure 3C). To investigate shifts in mitochondria-associated proteins within lung tissues, immunohistochemistry was employed. The results demonstrated a marked decrease in the expression of the mitochondrial fusion protein OPA1 and the mitochondrial biogenesis regulator NRF1 ( *P*< 0.05) following O _3_ exposure ( Figure 3D). However, the expression of the mitochondrial fission protein DRP1 was increased ( Figure 3E). These data indicate that O _3_ exposure damages the oxidative balance and mitochondrial dynamics in lung tissue. The reduction in SOD activity and increase in MDA levels suggest elevated oxidative stress, while the diminished activity of Na ^+^-K ^+^-ATPase indicates impaired energy metabolism. Furthermore, the downregulation of OPA1 and NRF1, alongside the upregulation of DRP1, implies a shift towards increased mitochondrial fusion/fission and biogenesis, contributing to mitochondrial homeostasis imbalance after O _3_ exposure.

### Dysregulation of homeostatic balance in AM mitochondria after O _3_ exposure

**Figure 4:**
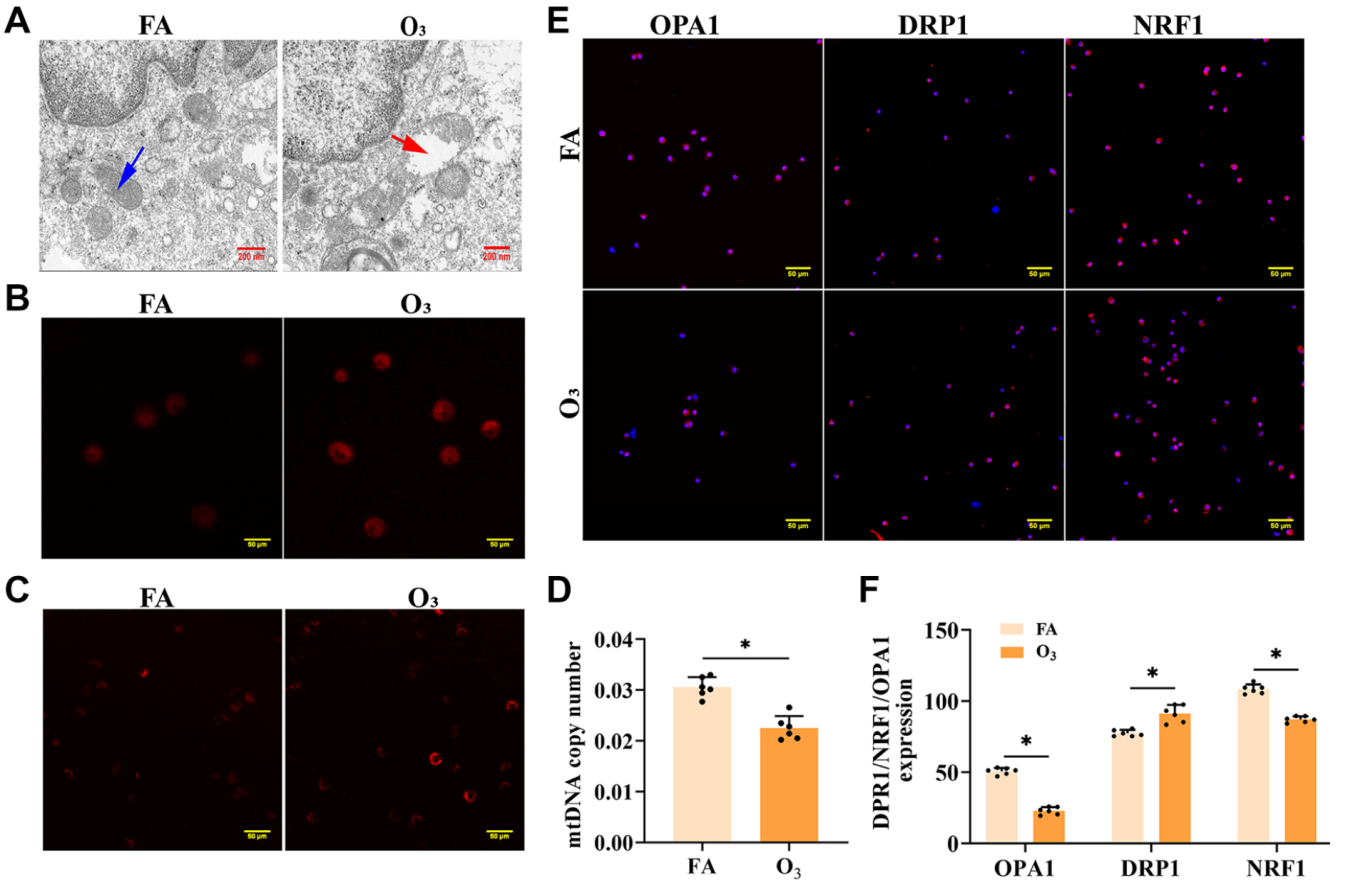
Figure 4 Mitochondrial structure, split fusion proteins and mtDNA alterations in AMs of mice after O _3_ exposure (A) Mitochondrial structure in AMs observed via electron microscopy. Blue arrow: normal mitochondrion; red arrow: mitochondria are markedly swollen, with matrix lysis and loss of cristae. Scale bar: 100 μm. (B) mtROS were detected by MitoSOX Red staining. Scale bar: 50 μm. (C) Mitochondrial quantity was determined via MitoTracker® Deep Red FM. Scale bar: 50 μm. (D) mtDNA copy number in AMs. (E,F) Expressions of mitochondrial fission/fusion-related proteins in the lung tissues of the mice detected via immunofluorescence staining were quantified via ImageJ software. Scale bar: 50 μm. *P < 0.05.

The structure of the mitochondria in AMs after exposure to O _3_ was studied in depth via transmission electron microscopy. Figure 4A shows structural variations, with the O _3_-exposed group displaying significant disruption of mitochondrial cristae characterized by fracture damage and matrix dissolution. Compared with those in the control group, the numbers of mitochondria and mtROS were significantly greater in the AMs of O _3_-exposed mice ( Figure 4B,C), and the number of mtDNA copies decreased ( Figure 4D). The expressions of mitochondrial fission/fusion-related proteins were altered, as evidenced by a significant increase in the expression of the mitochondrial fission protein DRP1 and a significant decrease in the expression of the mitochondrial fusion protein OPA1 and the mitochondrial biogenesis regulator NRF1 ( *P*< 0.05; Figure 4E,F). These data indicate that O _3_ exposure leads to mitochondrial homeostasis imbalance in AMs, marked by structural damage, increased mitochondrial number, and elevated mtROS levels. The reduction in mtDNA copies, along with the imbalance in fission and fusion proteins, suggests that O _3_ exposure promotes the increase of mitochondrial number and impairs mitochondrial biogenesis.

### Acute inflammatory injury is alleviated in NLRP3 ^‒/‒^ and Caspase-1 ^‒/‒^ mice after O _3_ exposure

**Figure 5:**
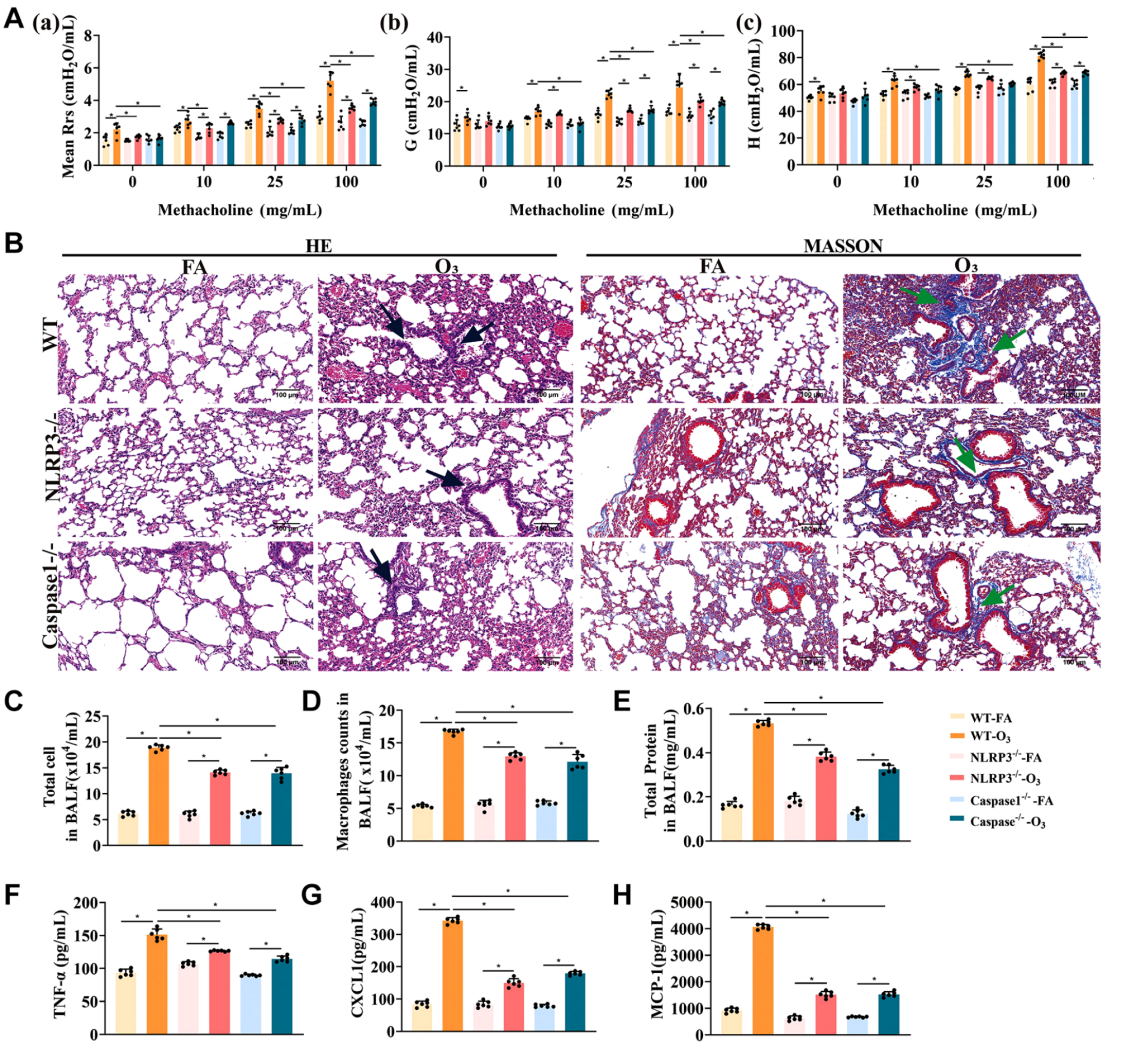
Figure 5 Acute inflammatory injury is alleviated in NLRP3 ^‒/‒^ and Caspase-1 ^‒/‒^ mice after O _3_ exposure (A) Evaluation of airway resistance (a), tissue damping (b) and tissue elastance (c). (B) HE and MT staining of the lung tissue of the mice. Bronchial epithelial inflammatory cell infiltration and thickening are indicated by black arrows, and collagen deposition is indicated by green arrows. Scale bar: 100 μm. (C) Total cell count in the BALF. (D) Total alveolar macrophage count in the BALF. (E) Total protein level in the BALF. (F‒H) Protein levels of TNF-α, CXCL1, and MCP-1 in the BALF of the mice. *P < 0.05.

The roles of NLRP3 and Caspase1 in O _3_-induced acute lung injury were assessed by measuring AHR and indices of lung inflammation. As depicted in Figure 5A‒C, knockdown of *NLRP3* and *Caspase1* mitigated Rrs, G, and H injuries in O _3_-exposed mice, along with reductions in inflammatory cell infiltration, bronchial wall thickening, and collagen deposition ( Figure 5A,B). Furthermore, *NLRP3* and *Caspase1* knockdown led to decreases in the total cell count, alveolar macrophage number, and total protein concentration ( Figure 5C‒E). Additionally, the protein levels of TNF-α, CXCL1, and MCP-1 were decreased ( Figure 5F‒H). In summary, these findings suggest that the knockdown of *NLRP3* and *Caspase-1* alleviates O _3_-induced airway hyperresponsiveness and lung inflammation.

### The NLRP3 inflammasome regulates mitochondrial homeostasis in AMs

**Figure 6:**
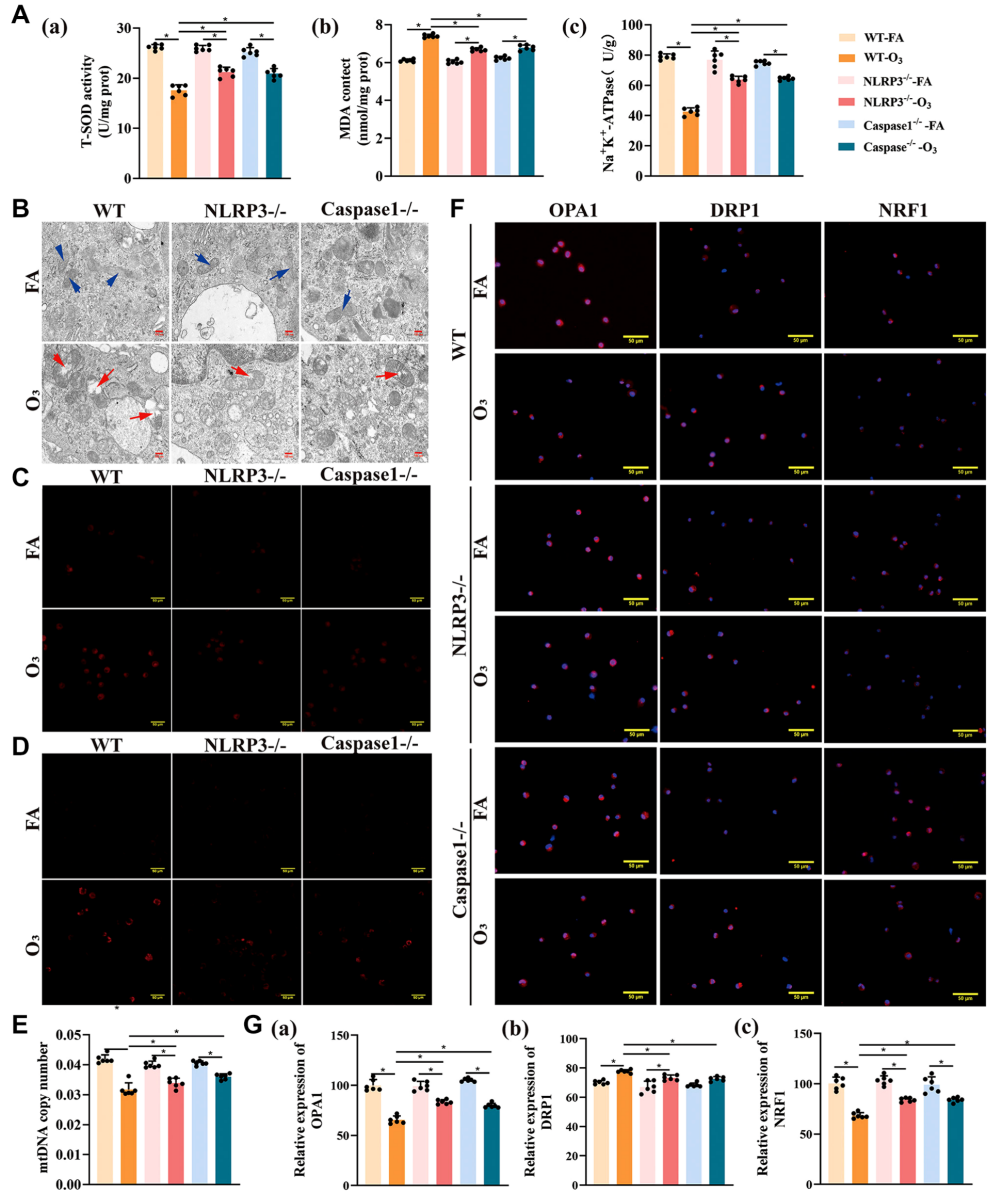
Figure 6 The NLRP3 inflammasome regulates mitochondrial homeostasis in AMs (A) Measurement of SOD, MDA and Na+-K+-ATPase levels. (B) Mitochondrial structure in AMs observed via electron microscopy. Blue arrow: normal mitochondrion; red arrow: mitochondria are markedly swollen, with matrix lysis and loss of cristae. Scale bar: 100 μm. (C) mtROS were detected by MitoSOX Red staining. Scale bar: 50 μm. (D) Mitochondrial quantity was determined via MitoTracker® Deep Red FM. Scale bar: 50 μm. (E) mtDNA copy number in AMs. (F‒G) Expressions of mitochondrial fission/fusion-related proteins in the lung tissues of the mice detected via immunofluorescence staining were quantified via ImageJ software. Scale bar: 50 μm. *P < 0.05.

Knockdown of *NLRP3* and *Caspase-1* resulted in significant outcomes, including a notable increase in the SOD and Na ^+^ -K ^+^-ATPase levels in the lung tissue ( *P*< 0.05) and a significant decrease in the MDA levels, as depicted in Figure 6A. Mitochondrial morphology in AMs revealed a significant improvement in mitochondrial structure following O _3_ exposure ( Figure 6B). This improvement was marked by a substantial reduction in mitochondrial number and mtROS expression ( *P*< 0.05) and multiple increases in mtDNA copy number ( *P*< 0.05) in AMs from both NLRP3 ^‒/‒^ and Caspase-1 ^‒/‒^ mice ( Figure 6C‒E). An examination of mitochondrial fission/fusion-related protein and mRNA expression in AMs ( Figure 6F,G) indicated that the protein levels of the mitochondrial fission protein DRP1 were significantly lower in NLRP3 ^‒/‒^ and Caspase1 ^‒/‒^ mice than in WT mice and that the protein levels of OPA1 and NRF1 were significantly greater in these knockout mice than in WT mice.

## Discussion

In this study, we demonstrated that (1) ozone exposure causes acute lung injury and increases the number of AMs and the protein expression of the NLRP3 inflammasome complex in AMs; (2) ozone activates mitochondrial dynamics but depresses mitochondrial metabolism and biogenesis in AMs; and (3) the NLRP3 inflammasome-mediated imbalance of mitochondrial homeostasis in AMs is involved in O _3_-induced acute inflammatory lung injury.

After O _3_ exposure, the total number of cells, especially AMs, in the BALF significantly increased, and the levels of macrophage-secreted chemokines (MCP-1 and CXCL1) and AM-secreted cytokine (TNF-α) were increased, which is consistent with studies of O _3_-induced lung inflammation [27]. Furthermore, the expressions of NLRP3 and Caspase-1 in the AMs of O _3_-exposed mice were increased, indicating that the NLRP3 inflammasome in AMs may be involved in O _3_-induced acute lung inflammatory injury. However, the NLRP3 inflammasome in AMs plays different roles in different lung diseases. Research has demonstrated that NLRP3 inflammasome activation in mouse AMs is associated with mechanical ventilation-induced lung inflammation and injury [28] and with lung pathology in response to exposure to multiwalled carbon nanotube (MWCNT) nickel contamination [29], which is consistent with our results. However, in AMs of lung cancer patients, NLRP3 inflammasome activation is decreased, and IL-1β secretion is decreased [30]. A possible explanation for this discrepancy may be that tumors induce immunosuppression in the lung cancer microenvironment and that AMs in lung cancer patients, as tumor-associated macrophages, play a significant role in lung immunoregulation for tumor escape. In this study, NLRP3 inflammasome activation in AMs may have contributed to lung inflammation induced by O _3_.

Ozone can damage mitochondrial structure and function [31]; however, little information is available about the changes in mitochondria in AMs after O _3_ exposure. In this study, mitochondrial morphology in AMs was observed with matrix lysis and loss of cristae. The number of mitochondria was increased, resulting in decreased expression of the fusion-related protein OPA1 and increased expression of the fission-related protein DRP1 in AMs. These changes in indicators in AMs suggest dysfunctional mitochondrial dynamics, which contribute to acute lung injury *in vitro* and *in vivo* [32]. It has been reported that downregulation of the OPA1 protein can promote the release of the chemokine CXCL1, leading to lung cellular senescence [33], which may explain why CXCL1 was elevated after O _3_ exposure in the present study. The accumulation of mitochondrial fusion could increase oxidative stress (ROS, lipid peroxidation) [34]. In this study, mtROS in AMs were also increased, which is very consistent with our findings. The accumulation of ROS in cells causes cellular dysfunction and the activation of the NF-κB complex to produce proinflammatory cytokines (TNF-α, IL-1β, *etc*.) [35], and ROS in cells oxidize cell membrane components, allowing the leakage of inflammatory factors from cells and worsening acute inflammatory injury [36]. In our study, TNF-α was also upregulated in the BALF.

Recent reports have shown that mitochondrial metabolic reprogramming is a driver of idiopathic pulmonary fibrosis [37] and that mitochondrial biogenesis dysfunction is often involved in the development of lung diseases, such as chronic obstructive pulmonary disease (COPD) and lung cancer [38]. In this study, decreased Na ^+^ -K ^+^-ATPase, which is associated with mitochondrial metabolism, in lung tissue was detected, and the levels of the mitochondrial biogenesis-related indicators mtDNA and NRF1 were decreased in AMs of O _3_-exposed mice, which suggests that ozone depresses mitochondrial energy metabolism and biogenesis. This finding is consistent with a study showing that sulfur dioxide (SO _2_) inhibits the expression of mtDNA and three factors of mitochondrial biogenesis, PGC-1α, NRF1, and TFAM, in lung tissue [39]. We inferred that the increased number of mitochondria in AMs may be from the fission of old mitochondria by mitochondrial dynamics but not new mitochondria by mitochondrial biogenesis. In the present study, the decrease in mitochondrial metabolism and biogenesis in AMs may be associated with AM dysfunction, which contributes to O _3_-induced acute inflammation. We speculate that the reasons may be not only that mtDNA and NRF1 are involved in mitochondrial biogenesis but also that changes in mtDNA copy number can disrupt the electron transport chain, interfering with ATP and ROS [40]; NRF1 may affect the level of the macrophage-related chemokine MCP-1 [41]. However, the detailed mechanism needs further investigation.

Many studies have demonstrated that ROS [ 42– 44], ATP [45] and oxidized mtDNA fragments [ 46, 47], which are usually produced from mitochondria, can act as factors to activate the NLRP3 inflammasome and subsequently trigger an immune response or inflammation. However, the interaction between mitochondria and the NLRP3 inflammasome may be bidirectional. Zhang *et al*. [48] reported that the NLRP3 inflammasome mediates albumin-induced renal tubular injury via impaired mitochondrial function and morphology. In our study, to verify whether the NLRP3 inflammasome regulates mitochondria in AMs, we isolated AMs from NLRP3 ^‒/‒^ and Caspase-1 ^‒/‒^ mice after O _3_ exposure and found that, compared with those in O _3_-exposed WT mice, the indicators of mitochondrial homeostasis (mitochondrial dynamics, metabolism and biogenesis) were reversed in AMs from NLRP3 ^‒/‒^ and Caspase-1 ^‒/‒^ mice exposed to O3, which suggested that the NLRP3 inflammasome could regulate mitochondrial homeostasis. We speculated that when the protein expression of the NLRP3 inflammasome complex is dysregulated or when there is abnormal NLRP3 inflammasome activation, mitochondrial homeostasis imbalance may occur, resulting in cell dysfunction.

Moreover, acute lung inflammatory injury in NLRP3 ^‒/‒^ and Caspase-1 ^‒/‒^ mice was attenuated compared with that in WT mice after O _3_ exposure; for example, the levels of TNF-α, MCP-1 and CXCL1 in the BALF were decreased, and mtROS were also downregulated in the AMs of O _3_-exposed NLRP3 ^‒/‒^ and Caspase-1 ^‒/‒^ mice. On the basis of these results, we speculated that in AMs, NLRP3 inflammasome-induced imbalance in mitochondrial homeostasis could release much more inflammatory cytokines and chemokines (such as TNF-α, CXCL1 and MCP-1) and ROS, which are involved in lung inflammation induced by O _3_.

Nevertheless, there are several limitations in this study. On the one hand, ASC, a component of the NLRP3 inflammasome, has not been explored. On the other hand, indicators of mitochondrial homeostasis have not been fully and comprehensively detected, especially factors related to mitochondrial metabolism, which have been detected in lung tissue but not in AMs. In addition, we did not focus on the effects of interfering with changes in mitochondria-associated proteins on ozone-induced acute lung injury. More studies will be performed in the future.

In summary, this study demonstrated that the imbalance of the NLRP3 inflammasome-mediated mitochondrial homeostasis in alveolar macrophages contributes to ozone-induced acute lung inflammatory injury. The discovery of this novel mechanism provides potential insight for the prevention and treatment of ozone-induced acute lung inflammatory injury.

## Supplementary Data

Supplementary data is available at *Acta Biochimica et Biphysica Sinica* online.
